# Total knee arthroplasty in a patient with a fused ipsilateral hip

**DOI:** 10.1186/s13018-015-0271-z

**Published:** 2015-08-19

**Authors:** Kevin Koo, Khang Chiang Pang, Wilson Wang

**Affiliations:** Singapore General Hospital, Outram Road, Singapore, 169608 Singapore; National University Hospital, 5 Lower Kent Ridge Road, Singapore, 119074 Singapore; Department of Orthopaedic Surgery, Yong Loo Lin School of Medicine, National University of Singapore, 1E Kent Ridge Road, Singapore, 119228 Singapore

## Abstract

**Background:**

Many patients undergoing total knee replacement for joint degeneration may have cartilage wear in other joints and thus may already have had various other surgical procedures performed for these. To date, there is no data or description in the medical literature detailing how to perform a total knee replacement in a patient who previously underwent an ipsilateral hip fusion.

**Method:**

We describe how this is done in a patient who had her ipsilateral hip fused in 30° of flexion. This presents a surgical challenge because the knee can only be flexed to 70° if done in the conventional supine position. This not only makes exposure more difficult but can also lead to complications including component malpositioning and extensor mechanism problems, such as patellar tendon rupture. We present this case study and describe, with the aid of a series of intra-operative photographs, how this can be performed, with special focus on patient positioning to optimize knee exposure in a patient with a previous hip fusion.

**Results:**

The modifications made during surgery allowed the knee replacement to be carried out uneventfully. The patient recovered well and was able to return to her usual activities.

**Conclusion:**

We had to make various modifications to the intra-operative positioning in order to carry out the surgery. We hope these practical pointers will help clinicians faced with a similar situation in the future.

## Introduction

Total knee replacement is one of the most common and cost-effective medical interventions performed currently [1]. Many patients undergoing total knee replacement for joint degeneration may have cartilage wear in other joints [2] and thus may already have had various other surgical procedures performed for these. To date, there is no data or description in the medical literature detailing how to perform a total knee replacement in a patient who previously underwent an ipsilateral hip fusion. Our case report describes the surgical technique of how this can be performed, with special focus given to patient positioning to optimize knee exposure in such a patient.

## Case history

Our patient is a 67-year-old Asian female. She has a history of hyperlipidemia but no other medical co-morbidities. Her surgical history included a right hip fusion 28 years ago for fibrous ankylosis, for which the cause was uncertain.

She also underwent a right knee arthroscopic debridement and microfracture 3 years ago. Intra-operative findings then were extensive osteoarthritis and cartilage loss over the medial femoral condyle as well as the medial tibial plateau. There was also a complex degenerative tear of the medial meniscus, moderate patellofemoral osteoarthritis as well as mild lateral compartment degeneration.

Premorbidly, she was community ambulant without aids, independent in activities of daily living and maintained an active lifestyle, where dancing was her main pastime activity.

She presented this time with worsening right knee pain over the last 3 years. She described her pain as being mechanical in nature, preventing her from climbing up stairs, and limiting her walking distance to about 1 km at most. Her pain score was 7 out of 10.

## Physical examination

Her significant findings on clinical examination were those of a right hip fused in 30° of flexion and neutral abduction/adduction. She had a shortened right limb with a true limb length discrepancy of 3 cm (limb length measured from the anterior superior iliac spine to the medial malleolus on the right was 78 cm and on the left was 81 cm). However, she compensated by arching her lumbar spine into hyperlordosis to achieve an apparent full extension of her hip.

Her right knee range of motion was 5° to 110°, with no medial-lateral laxity. Crepitus was felt throughout her range of motion.

## Pre-operative radiographs

**Fig. 1:**
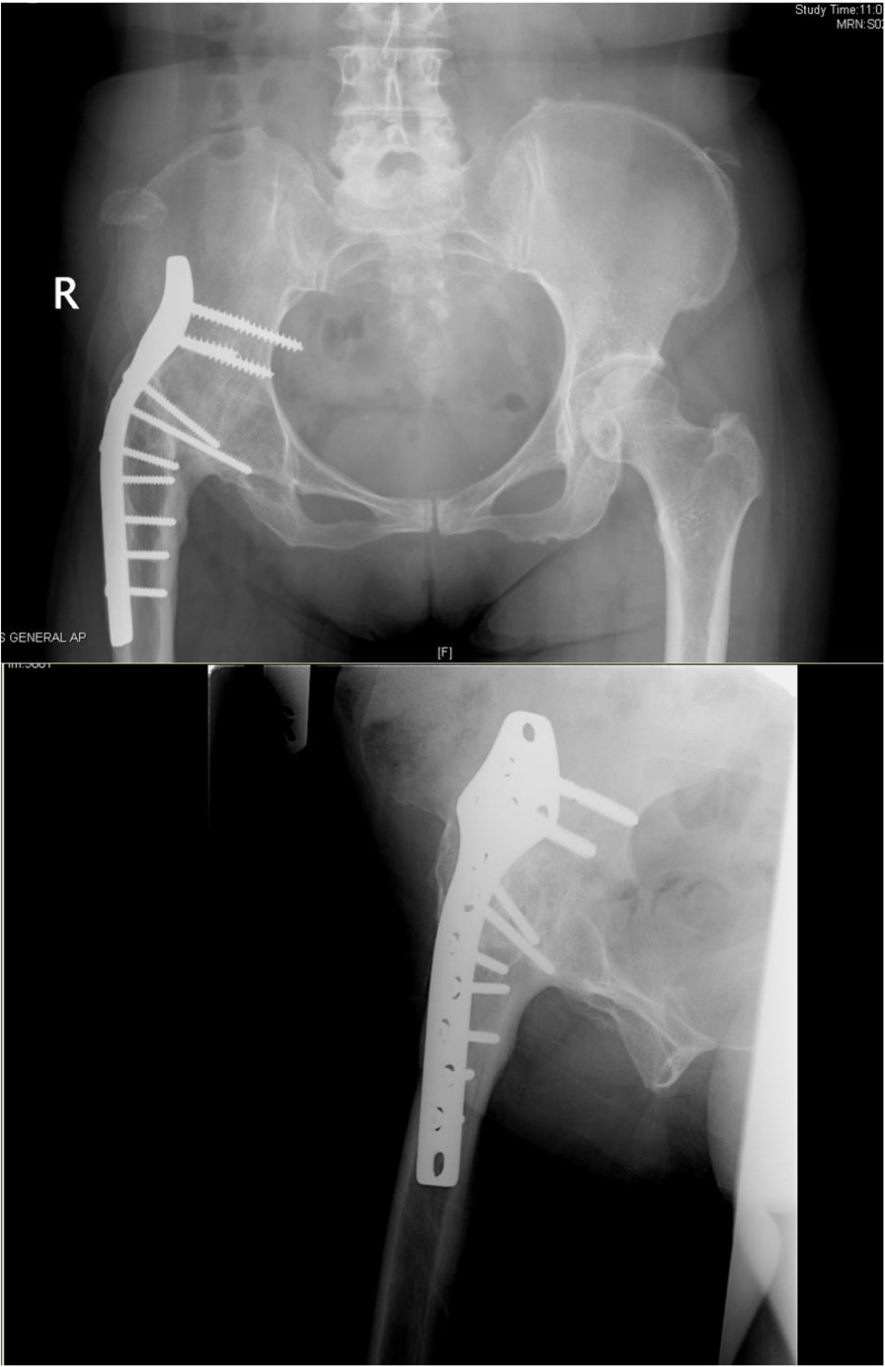
Existing hip fusion with a plate

Her pre-operative radiographs are as such. Figure 1 shows her existing hip fusion with a Synthes fusion plate.

**Fig. 2:**
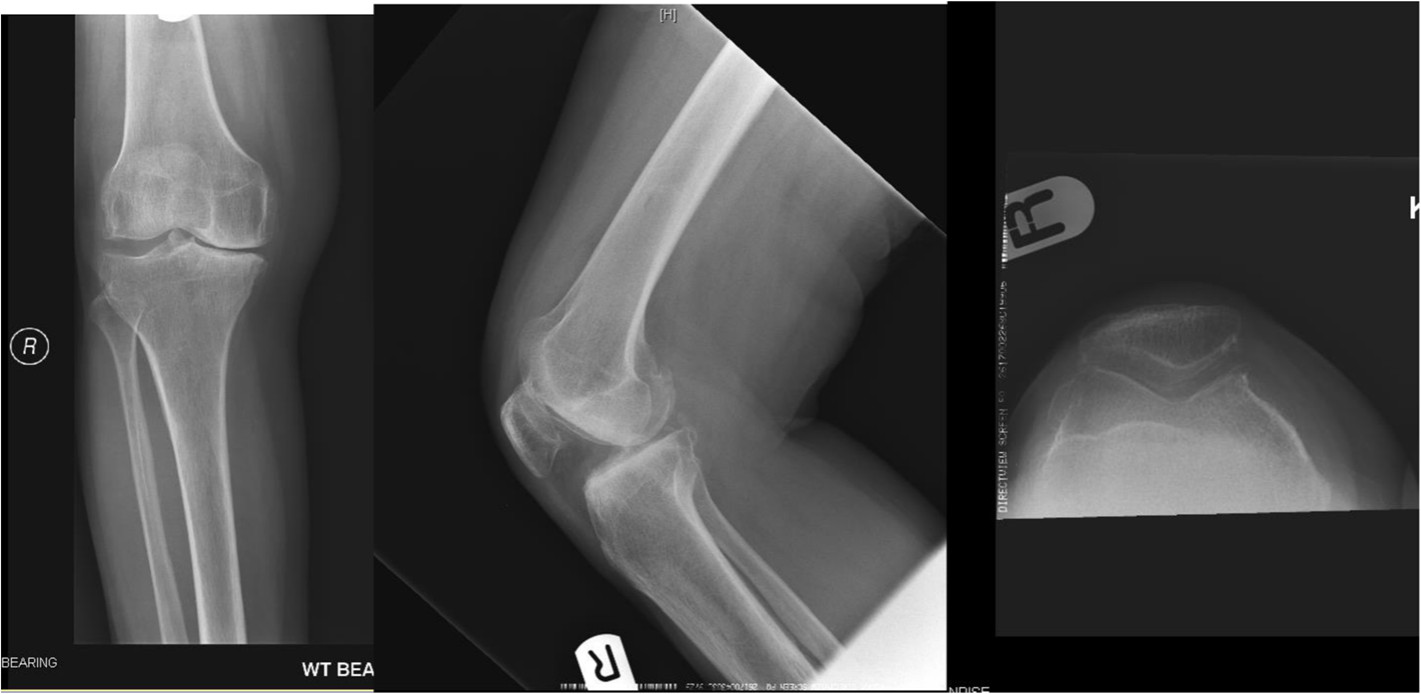
Pre-operative radiographs of her knees

Figure 2 shows the pre-operative state of her knees. Her right knee radiographs demonstrated tricompartmental degenerative changes, more severe over the medial and patellofemoral compartments, with mild genu varus.

## Methods (surgical technique)

The patient eventually underwent a right total knee replacement.

**Fig. 3:**
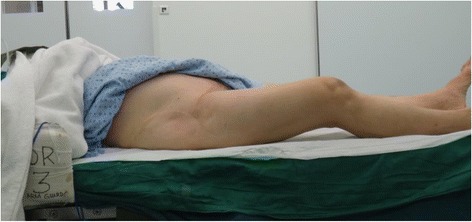
Position of her right lower limb, with her hip fused in 30° of flexion

Figure 3 shows the position of her right lower limb, with her hip fused in 30° of flexion.

**Fig. 4:**
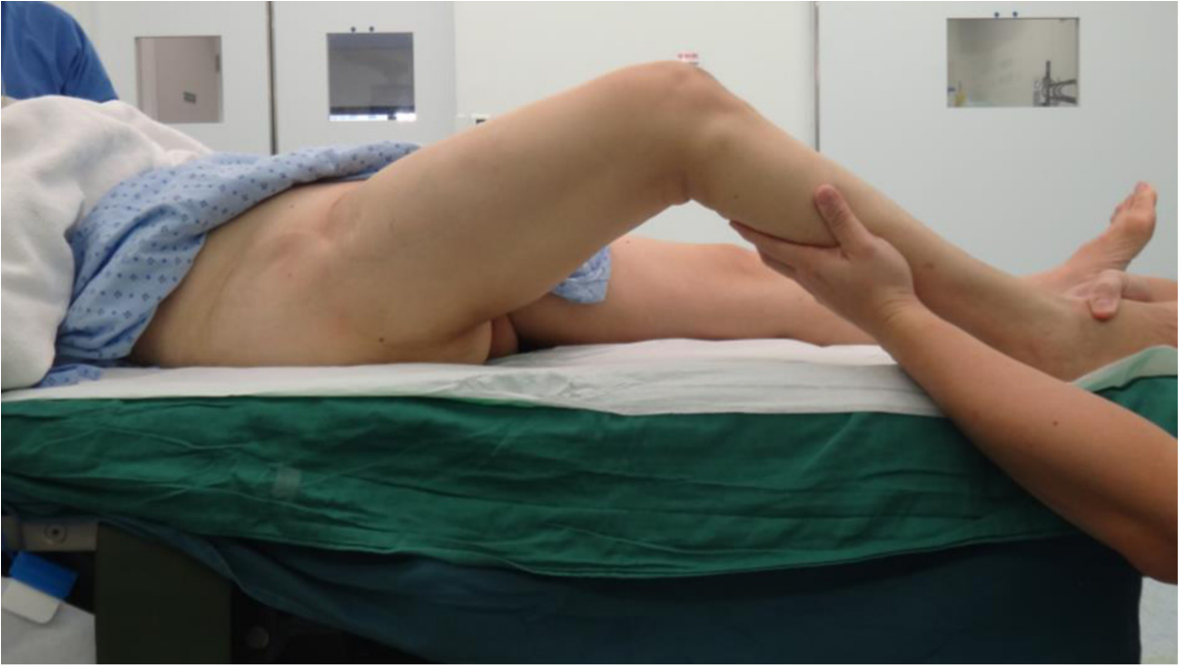
Knee can only be flexed to 70°

This presented as a surgical challenge in performing the knee replacement because the knee can only be flexed to 70° (Fig. 4) if done in the conventional supine position. This not only makes exposure more difficult [3] but can also lead to complications including component malpositioning and extensor mechanism problems, such as patellar tendon rupture [4].

**Fig. 5:**
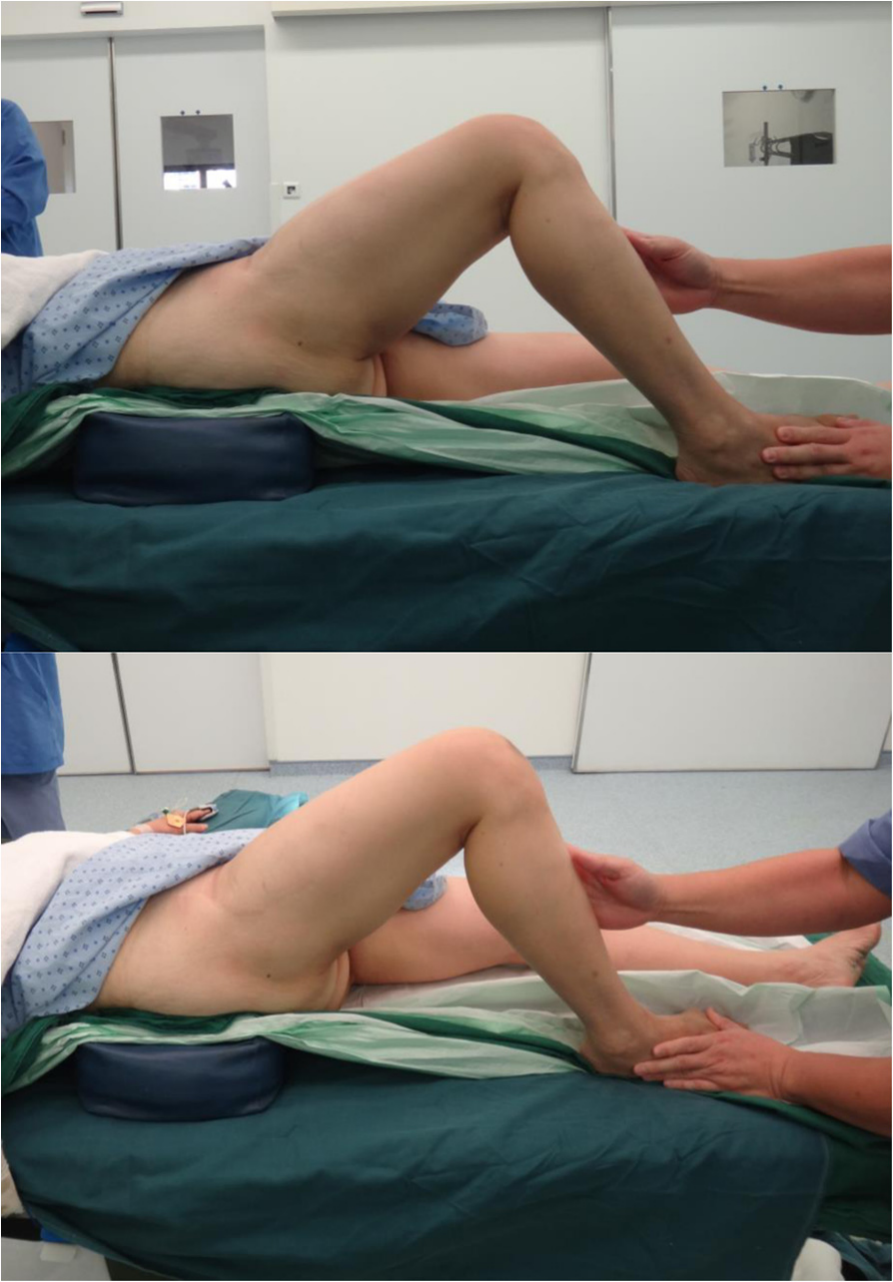
Modifications to the conventional way of positioning

We got around this problem by using a few modifications to the conventional way of positioning. Firstly, a sandbag was placed under the ipsilateral buttock. This elevated the fused hip joint and allowed the knee to flex up to 100° on the operating table (Fig. 5).

**Fig. 6:**
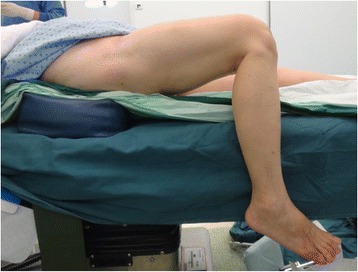
Contingency plan

A contingency plan, which we eventually did not have to use, was to hang the leg over the side of the operating table if more knee flexion was needed intra-operatively (Fig. 6).

**Fig. 7:**
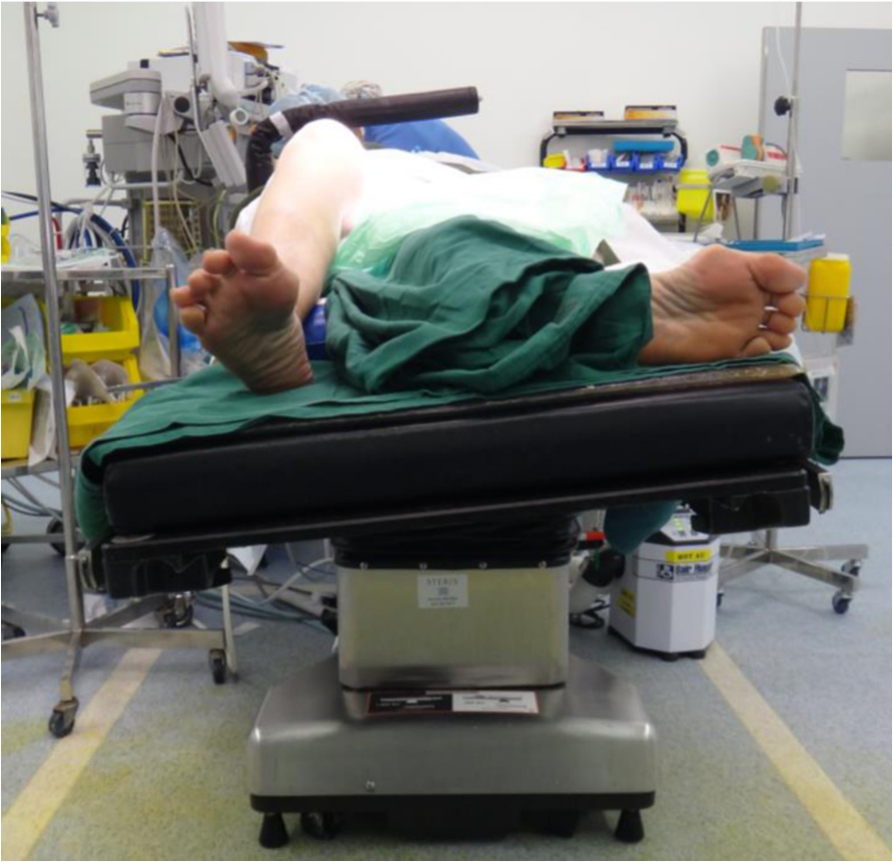
Operating table tilted towards the operated knee

Another modification we made was to the tilt of the operating table. By placing a sandbag under the ipsilateral hip, the knee will inevitable be forced into a tilt towards the opposite limb. To compensate for this, the operating table was tilted about 20° towards the operated knee, as shown in Fig. 7.

**Fig. 8:**
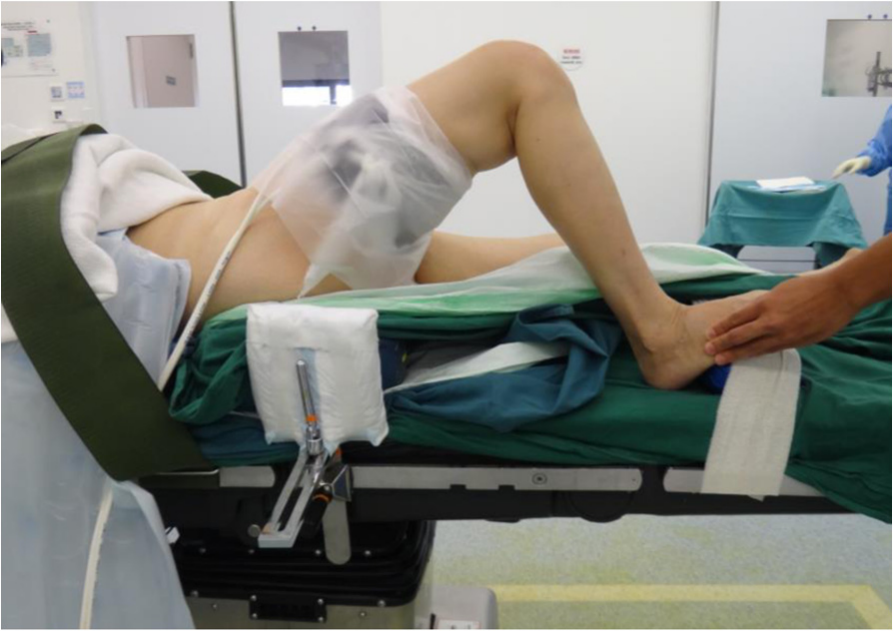
Final position before cleaning and draping

Figure 8 shows the final positioning of the patient just before cleaning and draping, with the knee achieving a flexion of 100°.

**Fig. 9:**
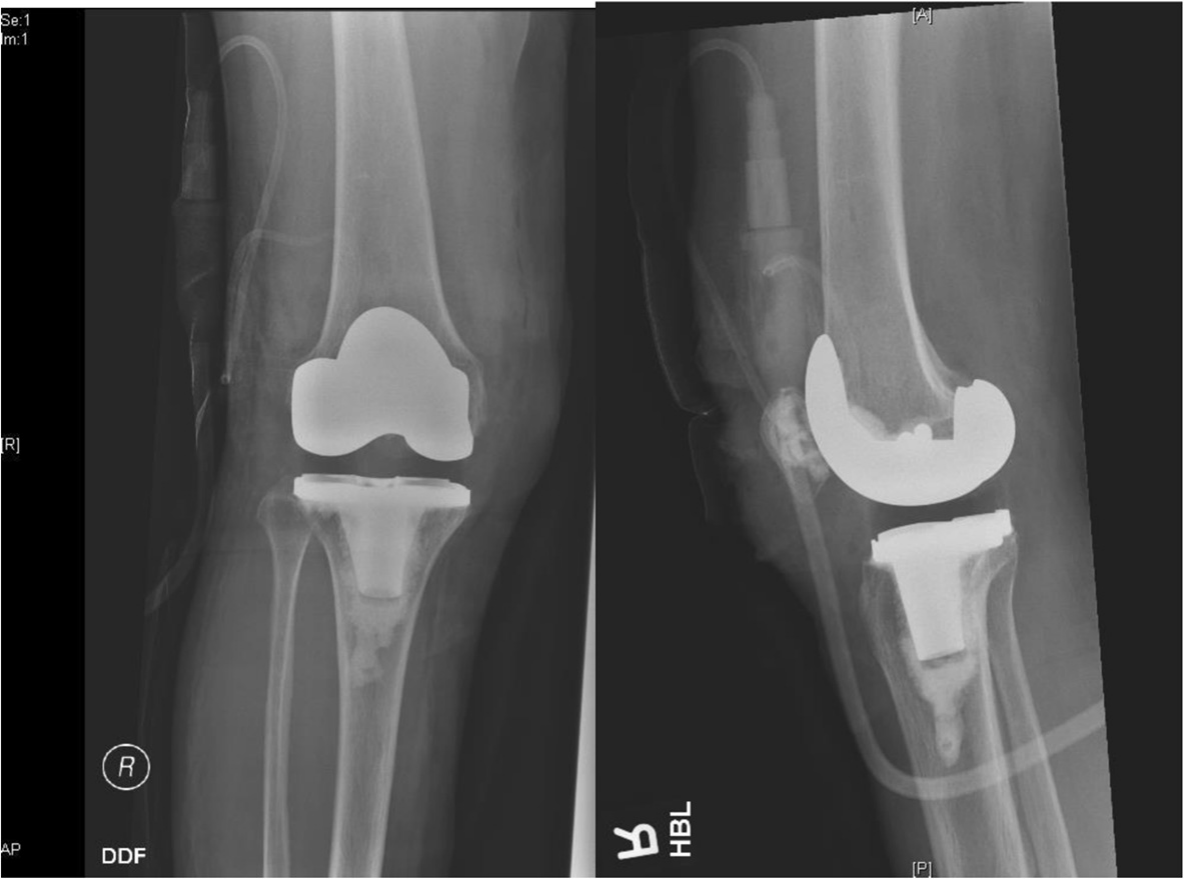
Post-operative check radiographs

The surgery proceeded uneventfully in this position. A posterior-stabilizing implant was used. Intra-operative finding was that of tricompartmental osteoarthritis. Post-operative radiographs showed a well-positioned implant (Fig. 9).

Approval for the study was sought and given by the institutional ethical review board (National Healthcare Group Domain Specific Review Board Ref: 2013/00514).

## Results

The patient recovered well in the ward post-operatively. She was able to ambulate on the 2nd post-operative day and was discharged on the 4th post-operative day.

At 6 weeks post-surgery, she was ambulating well, and her right knee range of motion was 0° to 95°.

At 6 months post-surgery, she was back to full activities except for dancing, which she attributed to a lack of time rather than function. Her range of motion was 0° to 120°, with a WOMAC score of 98.

## Discussion

Bonutti et al. [5] reported in their review of minimally invasive approach for total knee arthroplasty that decreased exposure to the knee can lead to various complications such as potential tibia component loosening. We feel that this can be a problem as well in doing a knee replacement in a patient with a pre-existing ipsilateral hip fusion if proper positioning is not done. Without adequate knee flexion, the patella may not be everted and thus compromise exposure to the joint.

Romness et al. [6] reviewed a series of 16 total knee arthroplasties done in patients with a prior ipsilateral arthrodesis or ankylosis. While they concluded that takedown of the hip fusion and conversion to total hip arthroplasty was an effective technique before performing the knee replacement, the numbers were too small to show that this was superior to performing a total knee replacement in a patient with an existing hip fusion, if the fusion was in a good position. There was, however, no description in this series of how the challenges in positioning were overcome in performing knee replacements in patients with existing ipsilateral hip fusions.

While the authors of that study reasoned that the position of the hip fusion, if suboptimal, can increase the stress on the knee implant and interface after replacement, thus favouring a conversion to hip replacement before knee surgery, they did not have sufficient numbers to back their claims and admitted it as a limitation in their study. In any case, our case report does not attempt to argue either way, but rather, it attempts to address the issue that if, indeed, a decision has already been made not to convert the fused hip to a mobile one prior to knee replacement surgery, then how best can we overcome the intra-operative technical challenges that come with that scenario.

## Conclusion

We have presented a rare case report of performing a total knee arthroplasty in a previously fused ipsilateral hip. There is currently no information in the existing literature on how this can be done. We had to make various modifications to the intra-operative positioning in order to carry out the surgery. This included placing a sandbag under the hip, tilting the operating table and hanging the leg over the side in order to achieve a degree of knee flexion necessary to carry out the surgery safely. We hope these practical pointers will help clinicians when faced with a similar situation in the future.

## Consent to publish

Prior consent has been obtained from the patient for all relevant data to be published.
